# Next-Generation Wearable Biosensors Developed with Flexible Bio-Chips

**DOI:** 10.3390/mi12010064

**Published:** 2021-01-07

**Authors:** Dahyun Nam, Jae Min Cha, Kiwon Park

**Affiliations:** Department of Mechatronics Engineering, Incheon National University, Incheon 22012, Korea; dorothy0639@inu.ac.kr

**Keywords:** biosensor, wearable biosensor, electrophysiology sensor, motion artifact noise, flexible electrode

## Abstract

The development of biosensors that measure various biosignals from our body is an indispensable research field for health monitoring. In recent years, as the demand to monitor the health conditions of individuals in real time have increased, wearable-type biosensors have received more attention as an alternative to laboratory equipment. These biosensors have been embedded into smart watches, clothes, and accessories to collect various biosignals in real time. Although wearable biosensors attached to the human body can conveniently collect biosignals, there are reliability issues due to noise generated in data collection. In order for wearable biosensors to be more widely used, the reliability of collected data should be improved. Research on flexible bio-chips in the field of material science and engineering might help develop new types of biosensors that resolve the issues of conventional wearable biosensors. Flexible bio-chips with higher precision can be used to collect various human data in academic research and in our daily lives. In this review, we present various types of conventional biosensors that have been used and discuss associated issues such as noise and inaccuracy. We then introduce recent studies on flexible bio-chips as a solution to these issues.

## 1. Introduction

With the spread of smart devices and increasing awareness of the importance of health, biosensors for simple and easy health management are receiving more attention. It is difficult to detect abnormalities in the body and diseases before they occur since the body consists of complex mechanisms that are not revealed. When an abnormality is felt, it may already be serious, which makes recovery difficult. Therefore, analysis of data obtained from the body is essential for a more convenient and healthy life. One of the methods for quickly finding abnormalities in the body is the use of a biosensor that detects biological information such as temperature and blood in the body. Biosensors that obtain biodata, which are information about the body, are currently being used for medical purposes such as independent health checkups and monitoring treatment progress by putting an analysis system inside the sensor. If a biosensor is developed in a wearable form for more convenient use, health monitoring can be applied not only in hospitals but also in daily life [1]. As the use of smart devices becomes more popular, the development of appropriate sensors is essential, as is the development of sensors applicable to the security and biomedical fields. 

The advancement of biosensors has brought about large differences in our lives. With the application of biosensors to medical devices, safer and more accurate diagnoses have become possible and biosensors have been applied in real life with the advent of data analysis technology such as big data [2]. With these developments, biosensors can be accessed in the form of smart devices that are most commonly seen in real life. For example, after exercise, oxygen saturation and heartrate can be easily measured with a smart device without going to a hospital or laboratory. However, there is a limitation in that measurement errors may occur due to poor contact and the surrounding environment [3]. For this reason, many biosensors have not been used in smart devices. To overcome these limitations, a broad understanding of biosensors is required. If we overcome the limitations and apply a wider range of biosensors to smart devices, there will be greater progress in terms of health monitoring. 

In this review, we suggest a method of measuring biosensors and the development process and limitations of these sensors for a broader understanding of biosensors. We hope that this will facilitate the development of systems that can be combined with biosensors or knowledge on how to blend biotechnology with what is currently being done. For this, it is essential to acquire knowledge about the sensor development process. The combination of biosensors and other technologies will contribute to the advancement of society, such as remote diagnosis and self-health checks. For that, the limitations of current biosensors and the development process are reviewed, and the direction of future progress is presented.

## 2. Current Biosensor Technologies 

### 2.1. Electromyography

**Figure 1:**
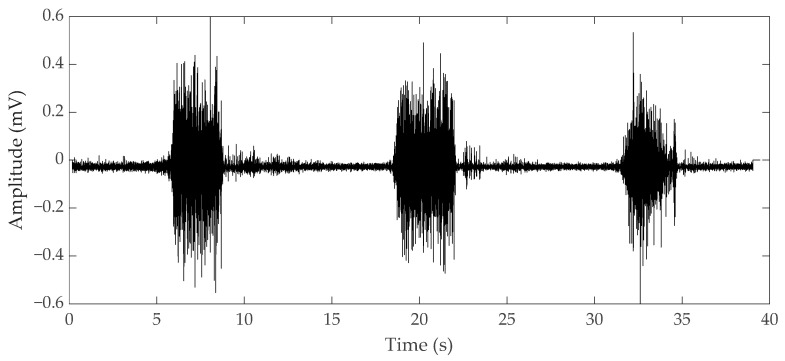
Electromyography (EMG) raw data during bicep brachii contraction and relaxation.

Electromyography (EMG) is a bioelectrical signal for in evaluating electrical activity produced by skeletal muscles. The EMG sensor detects the motor unit potential, which is a complex potential generated by the muscle fibers of the motor unit during spontaneous activity of muscle cells, enabling analysis of muscle activity. For muscles to work, electrical stimulation must be applied to muscle cells by nerve substances generated by a command from the central nervous system. Contraction and relaxation of muscles occur as a result of electrical stimulation, and these phenomena occur in several muscles, causing the body to move [4]. When our brain commands the muscles to contract, the central nervous system connected to the brain releases neurotransmitters and the neurons that receive these substances act to transmit electrical signals to the muscles, which make the body move. The electrical signals generated in muscle cells appear as mechanical signals, muscle contraction, and relaxation, and this transformation process is called excitation–contraction coupling [5]. The EMG sensor is a device that measures the electrical signals of muscles during this process. As an example of an EMG signal, the raw data when a muscle contracted and relaxed with a 1-kg dumbbell was measured by attaching an EMG sensor to the biceps brachii, as shown in Figure 1. When the muscle contracted, the value was larger than when the muscle was relaxed. Although the degree of muscle contraction can be visually confirmed with raw data, analysis is necessary for quantitative evaluation. EMG, which checks the degree of muscle contraction, is mainly used for medical and biomechanical research purposes [6]. For medical purposes, when movement is impossible for certain reasons, an EMG sensor is used to diagnose whether it is due to a problem in the nervous system or to damage to the muscle itself. When a muscle cannot contract or relax because of a muscle function problem, an electrical signal can be measured by EMG. If a problem occurs in the nervous system, the EMG signal cannot be measured. Also, it is possible to check the muscles that are activated during specific movements and activities, and through this, it is possible to study more efficient movements and activities. With EMG, it is also possible to analyze muscle peripheral and central fatigue during specific movements. If the contractile force of the muscle decreases due to peripheral fatigue, fatigue can be measured by increasing the raw data of the EMG signal because more electrical signals are required to maintain the same force. As the speed at which action potentials and excitations are transmitted by central fatigue slows down, the conduction velocity, which is the rate at which electrochemical impulses propagate into the nerve pathways, also slows down. Muscular fatigue is a combination of two types of fatigue, and the EMG sensor can determine the overall fatigue level [7].

**Figure 2:**
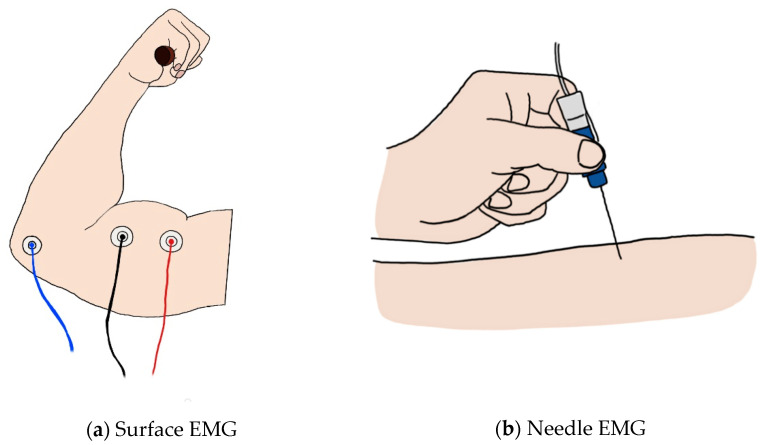
(**a**) Image of surface EMG: the red and black lines represent the + and − electrodes, and the blue line represents the ground; (**b**) image of the needle EMG.

EMG appears in two forms: surface EMG (sEMG), a noninvasive measurement method, and needle EMG, an invasive method. (Figure 2) sEMG is used more widely because it has a great advantage in terms of stability. However, since needle EMG directly inserts a needle into the muscle, more accurate results can be obtained than with sEMG [8]. As a result of investigating the relationship between the trigger point and the central nervous system to find problems in muscle pain syndrome and of conducting research with EMG, the necessary EMG could not be seen with surface recording techniques [9,10,11]. Although there are limitations of sEMG in a specific field, the development of technology for sEMG is required for implementation in wearable sensors. Since the applicable fields differ depending on the type of EMG, we divided this section into needle and surface EMG to review the research on EMG currently underway. 

#### 2.1.1. Surface EMG

Surface EMG is the most widely known and used method in measuring muscle contraction. Electrical contributions made by the active motor units are measured in a noninvasive way on the skin using electrodes [7]. sEMG does not simply measure the electrical signals generated by the muscles; it represents the potential difference between two electrodes. If two electrodes are placed along muscle fibers at both ends of the motor for the muscle, the same muscle action potentials at both ends of the muscle are recorded at the same time, making it impossible to measure the potential difference. Therefore, the EMG signal is measured using a differential amplifier that rejects a common signal from both electrodes using two electrodes [12]. Since electrical signals exist even in muscles that are not in use, reference electrodes are required to exclude redundant signals. Finally, three electrodes must be attached to receive the EMG signal of one muscle. Through this process, sEMG can evaluate the contractile ability of one muscle from three signals.

In the early days of sEMG, it was used for biofeedback. For example, a study was conducted to reduce the frequency and severity of tension headaches through EMG biofeedback, and another study found that the resting level of frontalis EMG activity is higher in tension headache patients than in the general population. From this, it was argued that contraction headaches caused by constant contractions of the scalp and neck muscles could be relieved by learning to relax these muscles through biofeedback [13]. In another study, when biofeedback was given through computerized electromyographic evaluation of pelvic floor muscles, the subjective evaluation of pain decreased by 83% after 16 weeks and the resting tension level decreased by 68% [14]. However, in the above study, since only the amplitude of the simple EMG value was used to check the contraction degree of the muscle and proceeded with biofeedback, more detailed EMG parameters were studied to evaluate the muscle performance. 

**Figure 3:**
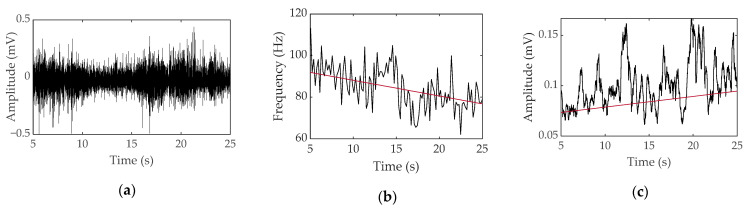
Surface EMG data obtained during voluntary contraction of a bicep with a 1-kg weight for a female: (**a**) raw data, (**b**) median frequency and linear regression, and (**c**) root mean square and linear regression.

To obtain the parameters, EMG data are mainly analyzed in two domains: a time domain where EMG data can be viewed over time and a frequency domain that can check the frequency of EMG data within a certain range. In the time domain, the degree of muscle activation can be visually confirmed over time. Because muscles require a larger electrical signal to generate more force, they exhibit a larger amplitude in the EMG data. Therefore, it is possible to check the degree of activation for each muscle using the root mean square (RMS) value [15,16]. Fate analysis in the time domain is also possible. When a muscle continuously exerts the same force, a larger electrical signal is required to maintain the same output; therefore, the more fatigue it builds up, the greater the amplitude displayed. Fatigue can be analyzed with an increase in RMS indicating an increase in amplitude [17]. In addition, various parameters such as integrated EMG and maximum amplitude are used in the time domain [18]. In the frequency domain, fatigue is mainly analyzed. As muscle fatigue builds up, the speed of transmitting electrical signals slows down and the frequency is lowered, so that the conduction velocity slows down [19]. To capture this, fatigue is quantified by using parameters such as mean frequency, median frequency, and peak frequency in a specific range [18]. An example of EMG parameters for fatigue analysis can be seen in Figure 3. 

Using these parameters, clinical studies have been conducted to measure muscle fatigue. Since the amplitude of EMG is different for each individual, fatigue was given using maximum voluntary contraction (MVC) and reliability was confirmed by analyzing the EMG parameters for fatigue (Table 1). Fatigue parameters were studied as RMS in the time domain, mean power frequency (MNF), and median power frequency (MDF) in the frequency domain, since the power spectrum frequency shift decreased. Mainly, the vastus lateralis (VL), rectus femoris (RF), vastus medialis (VM), lumbar 1–5, bicep brachii, and quadriceps were studied. Since the EMG signal has an accurate value when measured individually for one muscle rather than a complex signal of several muscles, it is limited to isometric contraction rather than dynamic contraction, which has a risk of complex measurement of several muscles. As a result of measuring EMG data during fatigue with isometric contraction, it was reported that most were reliable as indicators of fatigue, but due to the difficulty of application in dynamic contraction fatigue experiments and noise from electrode attachment, research on EMG continues. 

sEMG has a limitation in that it is a noninvasive method that is not directly inserted into the muscle. Because of the noise caused by the electrode, which is an attachment type, and due to the muscle attachment position of the electrode, it affects the conduction velocity and median frequency, parameters of EMG [27]. Standardization of the attachment method and attachment location per laboratory was required. Therefore, a study on standardizing the electrode position was conducted to obtain accurate and repeatable data on the sEMG signal parameters. Recommendations for the use and technical considerations of sEMG and a questionnaire and interpretations required for the use of sEMG were presented [6]. Several researchers studied the standardized electrode locations. For example, to provide information on the degree of uniformity of the inner zone position of 13 superficial muscles in the lower limb, an experiment was conducted and the optimal electrode placement was between the inner zone and the tendon termination according to the landmark in eight of 13 muscles [28]. This placement was suggested by examining the muscle fiber orientation and palpable bony landmarks in the abdominal muscle [29]. The surface EMG for a Noninvasive Assessment of Muscles (SENIAM) project standardized the electrode attachment location and electrode size for concerted action in the Biomedical Health and Research program of the European Union [30]. However, even if the standard electrode location is used, the biggest drawback of sEMG is not compensated. The limitations from previous studies are presented in Table 2. Some obtained the same results as the isometric contraction in dynamic contraction, and some did not obtain valid data. The studies that did not obtain valid data from the isometric contraction suggested that the reason was due to the movement of various muscles rather than one muscle when performing a specific movement.

When the EMG sensor is applied to a smart device, it is possible to recognize and prevent a muscle from being injured during exercise by monitoring the movement of the muscle. Since 70 years have passed since it was used for research, if a wearable sensor is made using the results of the research conducted so far, quality of life will be greatly improved. For example, there was a study that analyzed the frequency spectrum of electrocardiography (ECG) data that can detect EMG and EEG and can differentiate sleep states divided into four with 98–99% accuracy [37]. In addition, some studies have shown that driver fatigue can be measured by ECG and EMG [38,39]. In the case of drowsy driving, ECG and EMG can be developed for wearable biosensors to detect the risk of drowsiness at one of four levels and can greatly reduce accident rates. However, since sEMG and EEG need to have electrodes attached, they are inconvenient to wear and have not yet been commercialized due to noise problems. It was mainly analyzed only in the isometric contraction of a single muscle, and since dynamic movement appears when the movements of several muscles are integrated, it is difficult to measure accurate and significant data when performing dynamic movements. Also, a wireless sensor is essential for use outside the laboratory, such as running on a track or in a playground. Currently, a wireless sEMG sensor has been developed to analyze dynamic motion, but the noise that occurs in dynamic motion has not yet been resolved. For the EMG sensor to become a wearable sensor, it is necessary to solve the problem of noise generated on the surface electrode. The solution to this will be covered in detail in the section on noise below.

#### 2.1.2. Needle EMG

**Figure 4:**
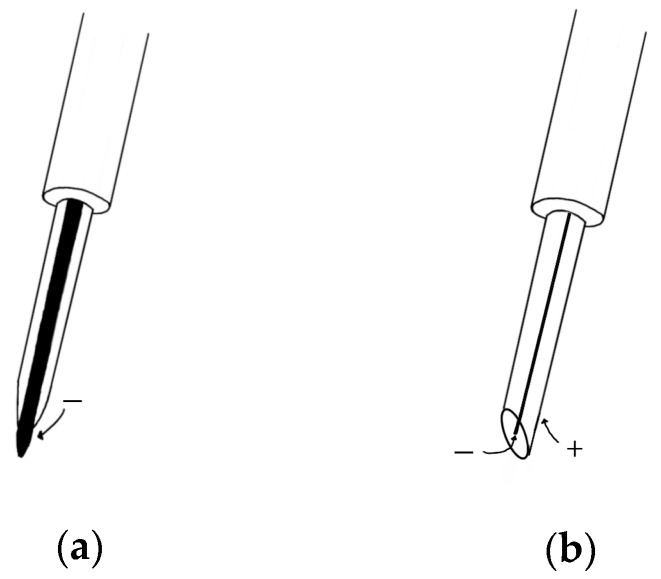
(**a**) Monopolar needle electrode; (**b**) concentric needle electrode.

Unlike surface EMG, needle EMG records the electrical activity of a muscle by inserting a needle electrode directly into the muscle or by obtaining the high-frequency electrical activity generated by moving the position of the needle electrode in the muscle and an electrical signal in a rest state [40]. The measurement of needle EMG by physical stimulation causes more severe pain than that of sEMG. In order to solve this problem, a study was conducted to investigate the pain of needle EMG [41], which can be divided into two types: a concentric needle electrode and a monopolar needle electrode (Figure 4). In the former, the surrounding cannula is the reference electrode, and the latter is recorded through the surface electrode. The electrode for measuring needle EMG is mainly a concentric needle electrode that has a small recording area and can obtain a value that cancels out noise. Since needle EMG can measure accurate data, one unit of motion can be analyzed through action potentials obtained from muscle fibers that contract at a location very close to the needle electrode using triggered averaging and decomposition methods [42]. After inserting a needle to induce minimal muscle contraction, the motion unit potential is analyzed by continuously obtaining one and the same motion unit potential using amplitude triggering while gradually moving the position. Muscles are evaluated mainly by comparing the average duration and amplitude with normal values [43]. After that, multi-motor unit potentials (MUP) analysis was developed and a technique capable of simultaneously obtaining and analyzing multiple-MUP from one recording site became available and was applied [44]. The pain of needle EMG, which was caused by the movement of the existing electrode, is less than in the past with multi-MUP analysis, which is possible with one insertion. However, there are still various limitations.

**Figure 5:**
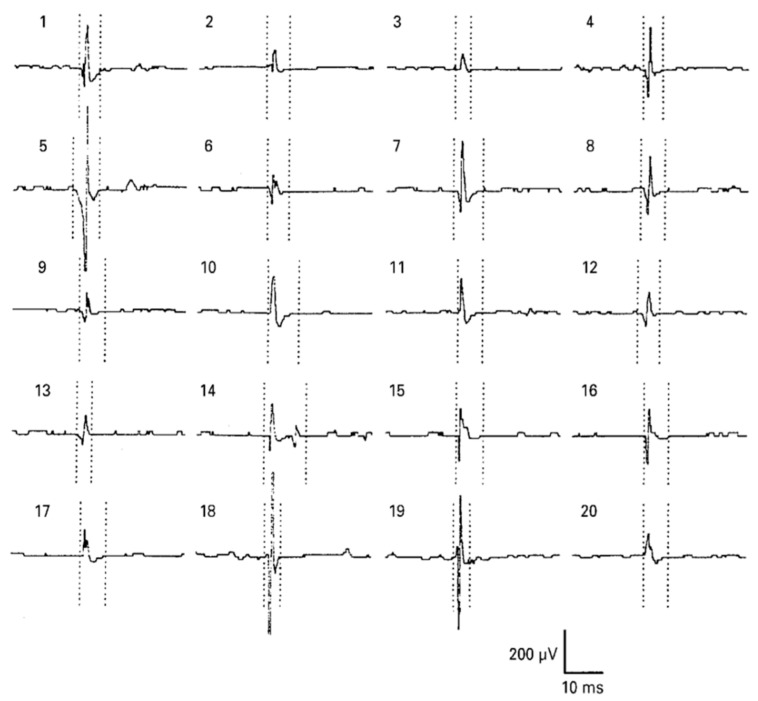
Needle EMG of the tongue: motor unit action potential (MUAP) from the right genioglossus muscle of a healthy subject. Markers for the MUAP duration were set on the most uncontaminated of the five averaged MUAPs. Reproduced from [50] with permission from BMJ Publishing Group Ltd.

Electrodiagnostic physicians should conduct a needle EMG test after checking the patient’s history based on clinical data. Care should be taken regarding some patients with skin infections, skin diseases, bleeding disorders, and obesity. Since there is a risk due to various variables such as muscle location and muscle size, needle EMG requires that the placement of a needle in the longitudinal midline of the muscle is accurately inserted into the muscle of interest. Instead of these drawbacks, it can be used as a measure of the accuracy of sEMG. For example, in the vastus intermedius muscle, sEMG was used to determine if the whole muscle could be used to assess neuromuscular activation. The observation of a good correlation could be used to assess total neuromuscular activation of the VL muscle during isometric contraction at low force levels [45]. There is also a paper on the feature extraction of a forearm EMG signal for prosthetics. EMG data analysis was conducted using both needle and sEMG for recording accuracy during hand movement [46]. For analysis of neuromuscular jitter that can be measured only in a single fiber, only needle EMG can be used [47,48,49] (Figure 5). EMG, which can obtain sensitive signals, is used for physiological analysis and diagnosis in a single motor unit during muscle contraction. Needle EMG is known to have a more accurate value than sEMG and is being used and studied. Needles have little noise, but considering the risk and difficulty of including them in a wearable sensor, it cannot be applied to the future development of biosensors. However, if the surface electrode is made in a way that reduces noise by bringing the reference electrode closer like a needle EMG to make it more accurate and easy to wear, it can be used in a wider range.

### 2.2. Electrocardiography

**Figure 6:**
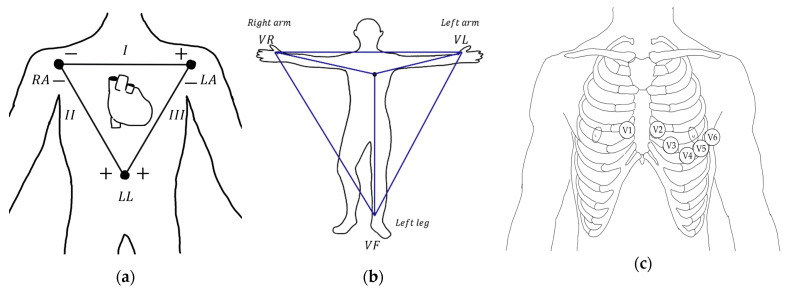
(**a**) Standard limb leads, (**b**) augmented limb leads, (**c**) precordial leads (V1: fourth intercostal space (ICS), right margin of the sternum; v2: fourth ICS along the left margin of the sternum; v4: fifth ICS, mid-clavicular line; v3: midway between v2 and v4; v5: fifth ICS, anterior axillary line (same level as v4); and v6: fifth ICS, mid-axillary line (same level as v4)).

Electrocardiography (ECG) is an interpretation of the heart’s electrical activity over a defined period. An electrocardiogram is used to measure the rate and consistency of a heartbeat and to diagnose the size, location, and condition of the heart. Since its introduction, it has become the most common clinical trial tool [51]. The voltage difference between myocardial cells, which is caused by depolarization of the myocardium whenever the heart beats, is detected and recorded. The ECG can be expressed as a cardiac vector, which is recorded by electrodes attached to the skin and by special equipment outside the body [52]. Over the past decades, advances in computers have made recommendations for the standardization of ECG records and interpretation of ECGs. For lead standardization, ECG is recorded through 12 leads composed of bipolar and unipolar leads [53]. There are three electrodes in standard limb leads, three electrodes in augmented limb leads, and six electrodes in precordial leads. Standard 1 is the left arm to the right arm, standard 2 is the left arm to the right leg, and standard 3 is the left arm to the left leg when using bipolar leads to obtain information about the electrical axis of the heart. Augmented limb leads is a method of recording signals attached to the limbs using unipolar leads. Augmented voltage right arm (aVR) is a method in which the right arm is positive and the average value of the left arm and left leg voltages is negative; augmented voltage left arm (aVL) is when the left arm is positive and the average value of the right arm and left leg voltage is negative; and augmented voltage left foot (aVF) is when the left leg is positive and the average value of the left arm and right arm voltage is negative. The precordial leads from V1 to V6 are attached to six areas close to the heart and receive signals using unipolar leads (Figure 6). Electrocardiogram electrodes, similar to sEMG, use a surface electrode where the skin and an adhesive pad are connected with a conductive gel, and skin preparation is required before attaching the electrode. Some ECG types, which require attention before measurement, are currently applied clinically and used for medical and research purposes.

With the introduction of X-rays, ECGs gave information about the structure and function of the heart. A galvanometer was used to record the difference between limbs due to electrical activation of the heart. After that, when the 12-lead ECG was invented, the ECG device we know was used in clinical and therapeutic applications [51]. With the development of the electrocardiogram, a study found that ventricular arrhythmias can stratify mortality risk in patients with ischemic stroke [54]. In addition, an independent risk factor for sudden death due to cardiac arrest was studied with ECG [55]. The health of elderly people without disease was monitored around the clock using ambulatory ECG, confirming that there was a significant prevalence of complex supraventricular beats [56]. Since it is not wireless and a professional person must be in the field, it is necessary to supplement it for application to a wearable sensor. It was confirmed that health monitoring is possible by using ECG wirelessly. Based on these advances, studies were conducted for the use of ECG in lifespan and health monitoring. Among them, the most interesting approach is the use of an electrocardiogram as an additional tool for biometric applications. It is the characteristic of the heartbeat based on the time and amplitude distances between detected reference points, and it can be used for identification with the human heartbeat [57,58]. However, since it is highly dependent on the accuracy of reference detection, it is necessary to accurately measure the ECG, and further advances in technology will be required to apply this to real life.

Since ECG, like sEMG, must use a surface electrode, accuracy is required when using the precordial leads method. Except for precordial leads, there is no problem with electrode placement presented in sEMG since it does not require precise positioning. ECG, which has fewer limitations than sEMG, was recently applied by mounting electrodes on the crystal and digital crown on the back of a smartwatch. Data was recorded using standard lead 1, a method of measuring the difference between the left arm and the right arm, and remote diagnosis was possible by connecting to a doctor. However, it is not possible to automatically measure for 24 h because a finger must be placed on the digital crown when measuring in everyday life. There is also concern that the data values may vary depending on the surrounding environment. Therefore, there are still limitations.

### 2.3. Photoplethysmography

**Figure 7:**
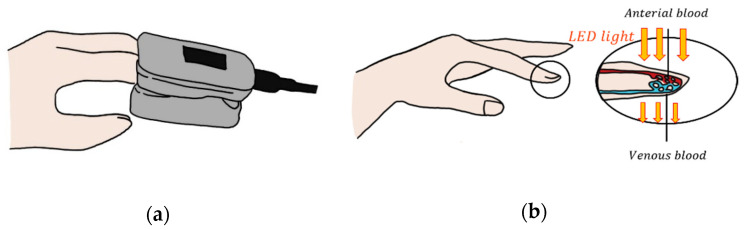
(**a**) The shape of a fingertip photoplethysmography (PPG); (**b**) transmitted absorption.

Photoplethysmography (PPG) detects blood volume changes caused by pressure pulses in the microvascular layer of photoplethysmogram tissue [59]. Changes in blood volume and flow are detected by measuring the amount of light transmitted or reflected through a photodiode by shining light on the skin from an LED. It uses the reflected light change as blood flow changes due to the action of the heart beating. Light from a source arrives at the finger and absorbs light from the blood, bones, and tissues, some of which pass through to reach the optical receiver and some of which are reflected. The degree to which light is absorbed is proportional to the amount of blood, skin, and tissue in the path through which the light passes, and since it is a component that does not change except for changes in blood flow due to heartbeat, it is possible to measure changes in blood flow [60]. With these properties, PPG can be used to measure and monitor breathing, hypovolemia, and other circulatory conditions [61]. PPG technology has been used in a variety of commercially available medical devices to measure oxygen saturation, blood pressure, and cardiac output; to assess autonomic function; and to detect peripheral vascular disease. PPG is used in smart devices to show the general process of heartrate. In general, PPG is measured at the fingertips using transmissive absorption (Figure 7). In the case of shock or hypothermia, it may not be properly measured. In this case, it is obtained from the ears, nose, and forehead by reflection.

The ancillary monitor of the “pulse waveform” PPG did not go through an intensive investigation. With the introduction of pulse oximetry to routine clinical practice in the 1980s, its importance in clinical medicine increased significantly. With the enhanced digital signaling technique, research is being conducted on parameters including the transmittance of light and reflection of light currently used in the field of PPG, such as waveform amplitude and rhythm [62]. Smart devices with PPG sensors that use the already certified transmittance and reflection of light have been applied and commercialized. Since PPG does not have a contact problem like that of a surface electrode, it is noninvasive and inexpensive and could be easily applied to a smartwatch using the method of measuring reflected light [63,64]. When ECG and heartrate variability were compared, PPG obtained meaningful similar results and was first used in smart devices because it is relatively cheaper than ECG. However, it has a limitation that it cannot see the heartbeat waveform like an ECG [65]. To further increase utilization of the PPG sensor, more research on PPG data is needed with the advancement of technology. Since the origin of the components of the PPG signal has not yet been accurately identified, studies are needed to demonstrate the usefulness of more than oxygen saturation and heartrate measurements in clinical settings. However, because PPG is sensitive to body movements, it is difficult to measure pulse rates precisely during exercise. A study was conducted to reduce the effect of motion artifacts generated by motion in a dynamic state [66,67,68]. Since PPG is a biosensor that is currently used as a wearable sensor, its stability is guaranteed, and if additional information about the waveform can be obtained, it will be a great improvement in the function of health monitoring.

### 2.4. Electroencephalography

**Figure 8:**
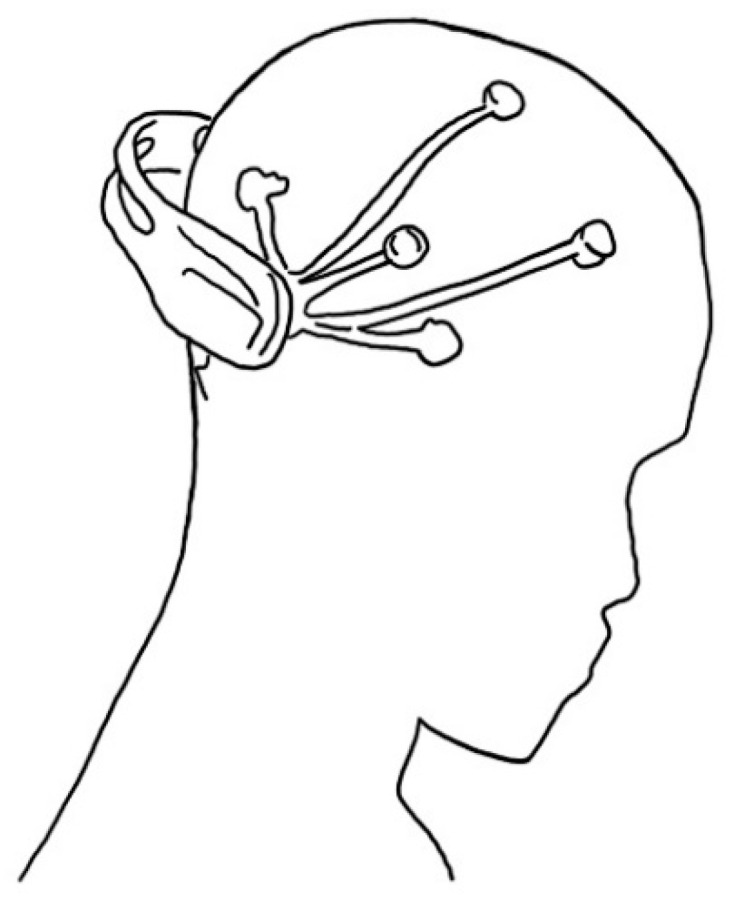
Head-mounted EEG device.

Electroencephalography (EEG) is an electrophysiological monitoring method that records the electrical activity of the brain [69]. In general, in clinical research and treatment, spontaneous electrical activity is recorded for a certain time using a noninvasive method placing electrodes along the scalp. The EEG provides information about brain activity by measuring the voltage displacement caused by ions within the brain’s neurons. Until the advent of MRI and CT, EEG was used for medical purposes to diagnose epilepsy, sleep disorders, depth of anesthesia, coma, and brain death [70]. Later, more advanced technologies such as MRI and CT were used in clinical research to monitor brain activity in real time or to study functions in neuroscience and cognitive science. EEG was also studied as a method to measure drive fatigue by investigating psychophysiological associations with fatigue. By looking at changes that occurred during the driver’s simulator operation, it was found that significant EEG changes occurred during fatigue [38]. Infants with encephalopathy and normal infants were studied within 12 h after birth for normal and abnormal patterns. As a result, infants with normal EEG findings were normal at follow-up at 18 to 24 months. Infants with moderately abnormal or suppressed EEG signals were confirmed to have a seizure or neurological abnormality [71]. In addition, studies on attention-deficit/hyperactivity disorder (AD/HD) were conducted with EEG. Since EEG is information on the background state of the brain that indicates cognition and behavior, studies have confirmed that EEG abnormalities are found with high probability as a result of investigating children with motor behavioral disorders using EEG [72]. Based on the research results of the EEG sensor, it was necessary to make a device to apply it. The surface electrodes with wires used in the laboratory cause discomfort when worn and limits the measurement location of EEG data. Since diagnosis with MRI and CT cannot be conducted with wearable sensors, EEG sensors are being developed in simpler wearable forms for early detection of diseases and health. EEG has evolved into a small head-mounted device that can record data for a long time at once (Figure 8). It was made by removing the wire connecting the electrode and the EEG recording device and by replacing it with a microchip including an amplifier and wireless transmitter mounted on the electrode itself. This EEG record can be analyzed using a computer or smart device [73]. However, there is no case where it has spread to daily life for monitoring purposes because of the inconvenience caused by wearing it on the head during use. Therefore, it has not been applied to health monitoring based on the data currently studied on EEG. For the use of ECG as a health monitoring function, there is also a problem with accuracy due to the movement of the sensor. Therefore, there is a need to propose an alternative with a next-generation biosensor that can maintain fixation and can remove inconvenience.

## 3. Limitations of Current Biosensors

Noise must absolutely be removed for a biosensor to be wearable. Researchers have considered data processing methods and filtering methods to eliminate noise, and they are still being studied, but we intend to present a solution that addresses the fundamental problem. Noise includes transducer noise and electrode motion artifact noise. These are the two types of noise and the direction of development required to eliminate them.

### 3.1. Transducer Noise

**Figure 9:**
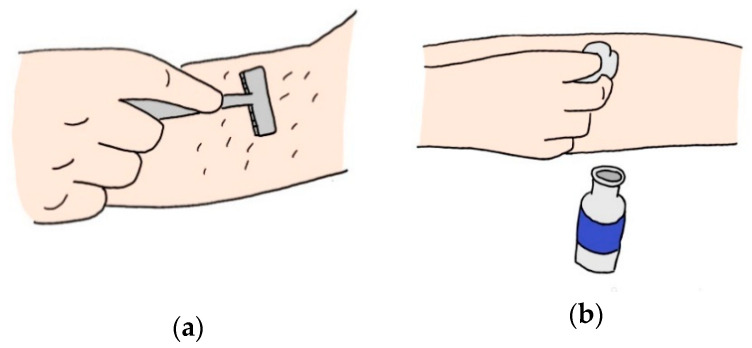
Preparations to reduce skin impedance: (**a**) shaving the attachment position; (**b**) disinfecting with an alcohol-soaked cotton.

Transducer noise occurs at the electrode-skin junction due to the difference in impedance between the skin and the electrode sensor, which is a conductive transducer. As skin impedance increases, distortion of the signal waveform and interference of the power line in the EMG occur. In order to reduce this skin impedance to an acceptable level, preparation of the skin is necessary before surface electrode application (Figure 9). The surface electrode mainly used has an electrolytic gel that minimizes skin impedance that generates noise, is relatively light, and can be manufactured in various sizes and shapes [74]. The gelled electrode serves to form a charge layer at the interface between the electrode metal and an electrolyte such as AG-AGCL. If the electrode is too small to use gel, a dry electrode is used. However, the skin impedance of the dry electrode is higher than the impedance of the gelled electrode [75]. Since the problem of noise occurs as a result of high impedance, an electrode circuit is required in the electrode part to minimize this. It is heavier than the gelled electrode, so it is difficult to fix the electrode. However, dry electrodes are mainly used in wireless sensors because amplification and filtering are possible at the electrode part without wires. In addition, since the dry electrode has more noise than a conventionally gelled electrode, more expensive special hardware is required [76]. The wireless sensor must have high accuracy to remove the limitation of motion. To reduce noise caused by contact with the skin, a sensor type that is less affected by environmental factors and minimizes impedance with the skin should be developed.

### 3.2. Electrode Artifacts during Body Movement

While measuring data through the surface electrode, the sensor and the skin should be in direct contact and the movement of the electrode should be minimized during measurement. If the sensor is not properly attached, correct data cannot be measured and incorrect data is provided [77]. In particular, the electrode is separated from the skin surface, creating a gap between the electrode and the skin during dynamic motion. In the case of a gel type electrode, as the intervening gel layer decreases, problems arise in measuring the electrical signal. Also, the skin is deformed or stretched, and the potential value changes during dynamic movement [78]. To solve these problems, skin impedance can be reduced by shaving the skin or by using a conductive paste. In the case of EMG, it is difficult to measure signals for only one muscle because EMG crosstalk from active muscles occurs in adjacent muscles during dynamic movement. Crosstalk is the biggest disadvantage of EMG signals and causes confusion in the interpretation of the results while recording on the skin surface.

## 4. Technology for the Next Generation of Flexible Wearable Biosensors

### 4.1. Noise Elimination

One countermeasure for noise is to decrease noise in the process of receiving data from the EMG sensor. Noise was eliminated by minimizing foreign matter such as hair and dead skin cells between the skin and the sensor for accurate signal measurement through the existing surface electrode. For more advanced technology, the processing method was studied in addition to the preparation for measurement. In EMGs contaminated with noise, interference was reduced by processing and filtering or noise was reduced using well-designed electrodes and signal recorders. For example, research has been conducted on algorithms and wavelets to eliminate powerline noise using signal processing techniques. By analyzing the ECG waveform with a high correlation value between the wavelet-processed signal and the original signal, it was proved that noise was diminished [79]. In addition, a study was conducted on a signal analysis method using parameters that are the least affected by noise among EMG signals. The study showed that a median frequency among EMG parameters has a very superior performance compared to other signals by adding white Gaussian noise to EMG signals [80]. Even though the existing parameters and sensor algorithms were improved, EMG was not able to study dynamic movements. In addition, even if noise is reduced, discomfort due to the fit of the sensor is an obstacle to the development of a wearable sensor. We present a new approach to this.

Despite various studies, it was not commercialized because of the quality problem of the sensor and the miniaturization of electronic devices. If a biosensor is used in textiles along with an electrode miniaturized by development of the sensor type, it is the next-generation biosensor we want. For the commercialization of various biosensors, it is important to minimize noise with technology that miniaturizes devices. If the noise problem is solved by approaches in various fields, advancement of the sensor could occur rapidly.

### 4.2. New Types of Biosensors

To improve the accuracy of the electrode and to create a sensor that is light and does not interfere with the fit, a new type of electrode may be combined with a biosensor. New types of electrodes are being studied that are more suitable for the body. For example, attempts were made to reduce noise by developing electrodes using fine needles or light and flexible materials. Since the existing gelled electrode cannot be used and noise is generated due to increased sensitivity to motion artifacts and poor skin-electrode contact, we came up with a textile sensor with a flex-printed electrode and integrated circuit [81,82]. If the biosensor type advances using microneedle patches or nanowires hybrid film, it will be possible to make a wearable sensor with high accuracy and minimal environmental impact by applying it to suits, shoes, and skin. Among the attempts to do this, a study was conducted on the development of a wearable ECG sensor to continuously monitor the ECG of children with a high risk of sudden infant death syndrome. Since the composition of circuits that communicate wirelessly, and amplify and filter wearable wireless sensors is essential, biosensors that are not uncomfortable to wear due to the application of various materials on the skin and that produce less noise as a result of external conditions have led to the use of various patches. In the method of applying the sensor to the fabric as a wearable sensor, if the clothes do not fit tightly, data errors occur. It is difficult to use in real life unless the clothes are tight enough to prevent discomfort. To solve this problem, it is necessary to strengthen the adhesion between the electrode and the skin so that it does not fall off.

Among the various types presented for the development of biosensors are micro-patches that deliver vaccines or drugs outside the skin. One of these is the microneedle patch, which performs injections and is attached to the patient’s skin through the microneedle. It was demonstrated that microneedles can penetrate the skin [83], and after that, microneedles were studied as a method for delivering drugs. However, we focus on the fixation power of the microneedles. So far, we have tried to make biosensors with safe, noninvasive methods for use in wearable forms. If you can minimize the invasiveness and make the biosensor light and convenient to operate, there is no need to insist on noninvasive methods. Since the stratum corneum of the skin is the largest barrier for drug delivery to the body, microneedles were developed to penetrate it and to deliver drugs in the stratum corneum of the skin without nerves. In clinical trials, microfabricated microneedles were proven to be painless when pressed on human skin [84]. The biggest reason for pursuing a noncontact method is the pain caused by needles entering the muscle. If a sensor is made in a wireless form using a microneedle patch, more accurate data can be received than a noninvasive method. Based on this, researchers recently developed electrodes through a microneedle. A microneedle array was used to develop wearable electrodes for biological signal recording [85] and to detect electrochemical and biological signals through blood. Microneedles have been studied for application ranges such as length, base diameter, thickness, and tip angle for clinical application [86].

**Figure 10:**
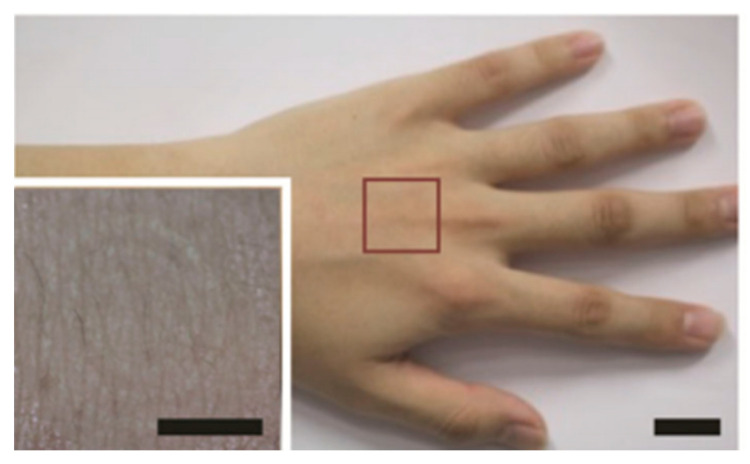
A transparent wireless sensor attached to human skin. Scale bar: 1 cm (inset: closeup image of the device. Scale bar: 0.5 cm) Figure reproduced with permission from Kim et al. [89]. Copyright Wiley-VCH GmbH.

Alternatively, electrodes are being developed with materials to modify the shape of light and flexible electronic products [87]. At present, the wireless biosensor has a limitations regarding the difficulty to measure in dynamic movement since circuits and dry electrodes for wireless communication are not able to deform and do not catch up with the deformation of the skin. If the biosensor is finely manufactured in the form of a microstructure and applied to integrated circuits, it will be possible to miniaturize biosensors and to produce them in flexible forms. Flexible sensors using graphene-nanowire hybrid nanostructures are being made this way. The transparent and highly stretchable nanowires hybrid film reduces the field-effect response and is advantageous for electrode applications. It has been shown that continuous connection to the channel can improve field-effect transistor mobility by reducing the contact resistance between the channel and the electrode [88]. As the technology for a transparent wireless sensor that can be attached to human skin is also advancing, it is possible to develop a bio-integrated wearable sensor [89] (Figure 10). A report on the electrical detection of immobilized proteins in a sensor with an AlGaN/GaN field effect transistor (FET) capable of lowering the noise ratio showed that the cell action potential can be recorded in cultured cells [90]. Thin films of indium tin oxide, a transparent conductive electrode material, were used, but there are limitations due to problems such as infrared transmittance and robustness. To replace this material, research has been conducted to apply carbon nanotubes and graphene. Among them, graphene, which was isolated by repeatedly peeling graphite with tape, has been recognized as a promising transparent conductor for electronic devices because of its high elasticity, low contact resistance, and structural stability [91]. There is also a study showing that graphene is capable of electrophysiological recording and optical imaging [92,93]. The use of metal nanowires to improve the transparency of biocompatible sheets has been introduced, bringing them closer to more advanced wearable sensors [94]. It is more conductive compared to materials, and since its effect is maintained even in a bent and elongated state, it reduces the discomfort of the wearer and helps in the application of a miniaturized wearable sensor. It was confirmed that the highly conductive Ag–Au nanocomposite can monitor electrophysiological signals along the skin contour [95]. With the use of various materials that can be implemented in the form of electrodes, it is possible to develop a sensor that is more flexible and suitable for movement of the human body. In order to reduce discomfort during wear, electrodes have been made with various materials to obtain more accurate values.

**Figure 11:**
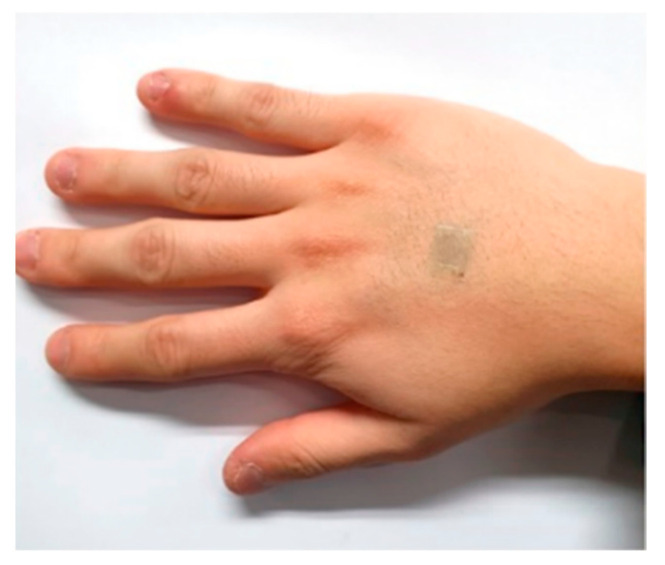
Transparent wireless heat patch. Reprinted with permission from Lee et al. [96]. Copyright © 2020. American Chemical Society.

As an example of the application of this material, sensor development in the temperature field is being made. Graphene has a higher thermal conductivity than metal and carbon nanotubes, enabling sensors using graphene to monitor temperature. A new approach to the temperature sensor was proposed with freestanding single-reaction graphene oxide fiber using the property where resistance depends on temperature [86]. In addition, studies were also conducted on skin heat patches and transparent supercapacitors and electrodes that embodied the heater’s function for heat treatment of skin tissue in the semiconductor film. For application to wearable sensors, transparent supercapacitors were used with electrohydrodynamic jet printing, and radio antennas and transparent and flexible electrodes were used in heat patches. Heat patches can be employed as a biosensor that can control the blood perfusion level and hyperfiction rate of the skin. Since noise generated in noninvasive data measurement can be minimized with low sheet retention, a method applying a dense AG nanofiber network was adopted (Figure 11) [96]. The graphene electronic tattoo (GET) format of graphene was proposed for bioelectrodes. Graphene bioelectrodes are highly likely to be applied to wearable electronic skin because they have high strain resistance and can obtain electrophysiological signals [92] Graphene is also a material of great interest for its application to biochemical sensors that can detect biodata from body fluids such as sweat, saliva, and tears. For example, using a contact lens sensor, we analyzed biodata obtained from tears in real time. There is also research that implemented contact lens sensors using RLC circuits [97]. Graphene has also been applied to sensors that identify human motion. A flexible and transparent strain sensor based on graphene and a carbon nanotube mixed film that can be worn has been reported [98]. A study in sports, a field that monitors human movement by attaching sensors to joints using graphene, a flexible and expandable material, was able to analyze the periodicity of joints and compared signals between joints to prove the stability of sensors [99]. Therefore, graphene can be applied as a transparent and flexible biosensor and has no resistance to attachment, so it can be accessed as a sensor and electrode in various fields. Although there have not been many clinical trials yet, commercialization can be achieved by combining the sensor’s data with a system that can be analyzed in real time.

**Figure 12:**
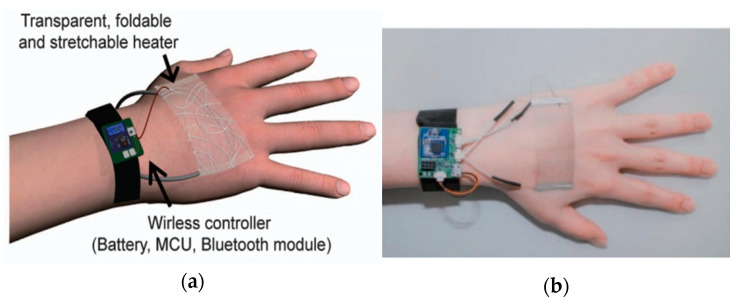
Transparent wireless heat patch with a microcontroller (MCU): (**a**) wireless controller and stretchable heater, and (**b**) a stretchable heater worn on the arm. Figure reproduced with permission from Jang et al. [100].

For real-time analysis by wearable sensors, a combination of a heat patch and microcontroller unit (MCU), which can send and receive data wirelessly, has been studied (Figure 12) [100]. In order to lessen inconvenience when attaching it, the circuit inside the electrode is not opened, the MCU is installed around the device, and the data received from the electrode is analyzed by the MCU to provide bio-signal information. As seen in Figure 12, it is composed of a wire-connected form, and if the technology is developed to miniaturize the sensor and circuit, it will be possible to manufacture the sensor in a form that is convenient to wear.

In another study, the use of inexpensive printed electrodes on flexible substrates such as plastic and paper was proposed. The screen-printing technique, which is used for printing on a thin surface, can be employed for electrochemical analysis by using ink with high printing viscosity [101]. Using the fast IR sintering method on a reusable coated paper substrate, it is possible to fabricate an electrode that has high conductivity and maintains conductivity while bending and twisting. This printing method has the advantage that the surface is smooth so there is no foreign-body feeling, and the material cost is low because it can be made thin. In terms of electrophysiology, direct attachment technologies such as inkjet printing have been suggested. Poly(3,4-ethylenedioxythiophene) polystyrene sulfonate was printed using inkjet technology for a conductive polymer to produce an electrode with good elasticity. An additional method of printing with ionic liquid gel was also used to improve contact with the skin. By measuring an ECG between two forearms through a printed electrode, it was found that essential electrocardiogram waves were clearly recorded [102]. There was also a study that realized an electrode for EEG, a sensor that measures electrophysiological signals similar to ECG as a flexible screen printed electrode. Results similar to those of general EEG were obtained by designing and attaching an Ag/AgCl electrode system printed on the auricle that can receive EEG signals. Nevertheless, since there was a problem caused by skin impedance in the existing EEG, further development of printed electrodes is required for complete noise reduction and comfort.

**Figure 13:**
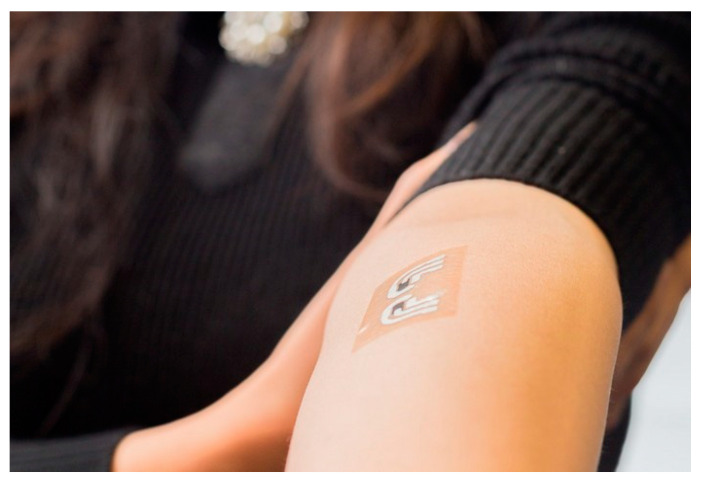
Epidermal electronic tattoo. Copyright © 2014. Jacobs School of Engineering/UC San Diego.

In a similar way, epidermal electronic systems (EES), which use a device with an mass density consistent with the epidermis, have been used to study the epidermal electrons that can detect biodata by coating the skin [103]. The device was coated on the skin and glued in a way that was mechanically invisible to users, like a tattoo (Figure 13), proving that data was available similar to that provided by game controllers [104,105]. An electrophysiological signal can be obtained by following the skin’s contoured surface and dynamic movement over time in a natural way that can be firmly integrated into the skin surface. After attaching the sheet to the skin and washing it off, the remaining EES on the skin adheres firmly. When this method is used, EES does not require a separate substrate, so it is appropriately attached to the human body and does not fall off easily, and it is possible to accurately measure electrical signals within the skin by naturally following the deformation of the skin. The question of how long EES is maintained in real situations was presented, and as a result of conducting an experiment by attaching it to the forearms of six participants, it was found that it could accommodate exercise, water, and sweat for 1 to 2 weeks [103]. One problem is that device failure is caused by exfoliation of dead skin cells. Although it is necessary to overcome these shortcomings for real-time monitoring of health, a method to apply the sensor most appropriately to the human body with the use of EES has been proposed. If the EES can be used once or for short intervals, such as disposable surface electrodes, real-time monitoring with wearable sensors will be possible.

If the combination of electrophysiological biosensors and devices with various materials is realized, it can be used to monitor and detect changes in our bodies in real time. The development of next-generation biosensors continues to be made with changes in electrodes, systems, and patches in various materials. A method to reduce noise will proceed in various directions with more studies, and further advances in the currently researched technology will continue to develop technology for health monitoring.

## 5. Concluding Remarks and Future Directions

**Figure 14:**
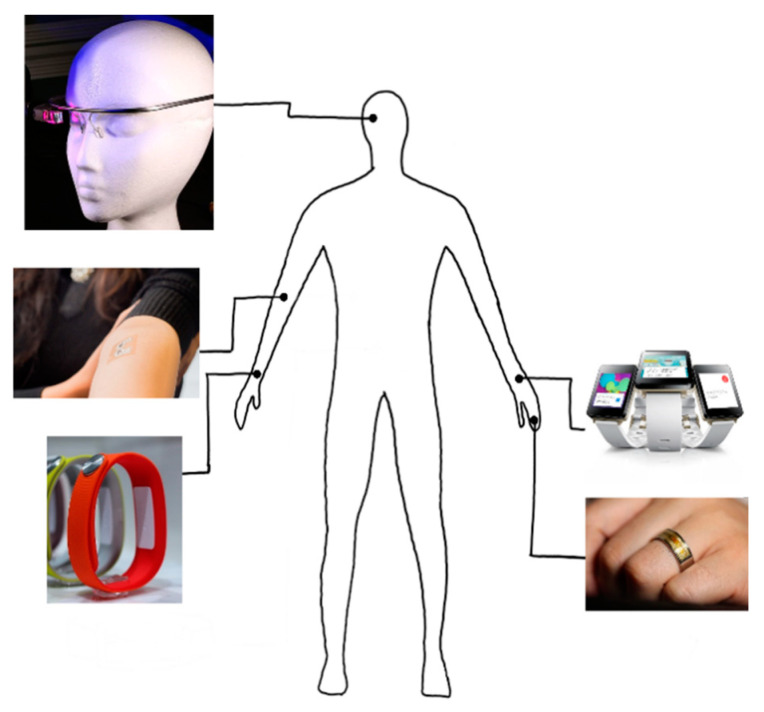
Wearable sensors.

Biosensors help to judge the state of our body by receiving signals from the body. They are used in smart devices that can be easily carried and are widely used in real life such as for health monitoring. The currently applied health monitoring functions include PPG and ECG. The use of a biosensor that we often see in smart devices is a health check function through heartrate measurement and oxygen saturation measurement using a PPG sensor. However, with a few exceptions, biosensors are not used in smart devices for some reason. The biggest reason is the noise caused by the surface electrode. Along with a method to minimize this noise, progress on wireless sensors is being made. Despite the development of wireless sensors, they have not been commercialized because of inconvenience when wearing them and are used only for research and medical purposes. An example of a wearable sensor can be seen in Figure 14. In order to solve the problem of adhesion of the surface electrode, noise generated by foreign substances such as sweat and movement during high-intensity exercise must be resolved. To address these problems, it is necessary to change the shape of the sensor to that of a microneedle patch.

For application to wearable sensors, studies on flexible sensors [106] and nonflexible sensors [107] are being conducted. If the microneedle is used together with a shape change of the flexible sensor, more accurate and painless measurement is possible. It does not fall off even if it moves dynamically, and it measure in places where measurement is restricted, such as in underwater, so clinical trials are possible in more cases. In the case of the dry electrode, when dynamic movement is performed, the skin is stretched and the adhesive of the sensor fails, making it difficult to analyze muscles. If a sensor is developed using microneedles, the fluidity of the flexible sensor does not degrade and more accurate data can be obtained. Since it can be measured closer than a conventional sensor, it will not be more sensitive to changes in the external environment. Also, if a printed circuit board (PCB) substrate can be used with a skin patch, it will be possible to achieve the smallest next-generation biosensor and wearable sensor that does not feel uncomfortable. As the current smart devices advance, more lightweight and miniaturized devices continue to be made. The combination with a microneedle is essential for the development of a biosensor based on painless, safer, and more accurate values with the integration of several technological advances.

A brief description of biosensors and the next-generation direction for development into wearable sensors was given. Biosensors are an indispensable field for future technologies as awareness of health has increased as we enter an aging society. For sudden cardiac arrest or remote monitoring of the elderly, a biosensor must be able to receive signals continuously for 24 h in real life [84]. In addition, an understanding of biosensors is essential for the early detection of diseases. The authors hope that, with this brief introduction to biosensors, they can be applied to each specialized field and developed in a wider variety of directions than the biosensor we proposed. If more data can be received through more biosensors and applied to smart devices, a more advanced life will be possible.

## Figures and Tables

**Table 1:** Reliability of EMG parameters for fatigue indices.

Ref	Purpose	Electrode Location	Subjects	Experimental Method	Parameters Used	Conclusion
[20] 2000	Investigation of EMG variable (MNF, RMS) for valid indicators of muscular fatigue	VL, RF, VM	11 males, 10 females	Repetitive maximum isokinetic knee extensions	RMS, MNF, MDF, torque, knee joint position	MNF is a good criterion validity.
[21] 1999	Investigation reliability of sEMG	VL, RF	9 males, 9 females	Isometric knee extension	RMS, MDF, torque	MVC measurement is best suited for clinical applications from rectus femoris muscle.
[22] 1999	Correlation of EMG fatigue data in the lower back to the subject’s assessment of fatigue	L1, L5	25 males, 25 females	Sørensen test	MDF, endurance time, Borg scale	The Borg scale correlated with endurance time and EMG median and mean power frequency slopes.
[23] 1982	Examination of the changes in frequency and amplitude of sEMG	Adductor pollicis, handgrip muscles, biceps, quadriceps	6 males	MVC, fatiguing contraction (25,40,70% MVC)	RMS, center frequency	The center frequency of sEMG appears to be a good noninvasive index of muscle fatigue.
[24] 1979	Study of quantitative changes in the EMG pattern muscle fiber-type distribution	VL	11 males	MV knee extensions	Integrated EMG, MNF	MNF decreases in FT-type muscles.
[25] 1986	Determination of the effects of motor unit recruitment and firing frequency on the surface EMG power spectra during sustained MVC and 50% MVC of the bicep brachii muscle	Bicep brachii	12 males	50% MVC	RMS, MNF	Increasing RMS EMG amplitude and decreasing MPF could better represent the MU activity during fatigue.
[26] 1994	Examination of the relationship between EMG manifestation of fatigue and endurance time during isometric contraction of the back extensors to fatigue	Erector spine at the levels of the 10th thoracic and 3rd lumbar vertebrae	21 males, 208 females	Sørensen test	MF, endurance time	MFgrad is a suitable technique for monitoring back muscle fatigue.

**Table 2:** Clinical studies conducted by measuring EMG during movement.

Ref	Purpose	Electrode Location	Subject	Experimental Method	Parameters Used	Conclusion
[31] 1993	Investigation of EMG median frequency of calf muscles during an exhausting treadmill exercise	Right soleus, gastrocnemius medialis, gastrocnemius lateralis	7 males, 2 females	Uphill treadmill run till the moment of exhaustion	Heartrate, ECG, median frequency,	Immediately after the run, isometric median power frequency declined.
[32] 2007	Determination if a difference existed in the rate of fatigue of select shoulder muscles during isometric shoulder elevation and if the measured rate of fatigue was consistent from day to day	Upper trapezius, middle deltoid, serratus anterior, lower trapezius muscles	7 males, 9 females ^­­­^	60% of their maximal voluntary isometric contraction force (MVIC)	MPF	Middle deltoid appears to fatigue faster than the other shoulder muscles tested at the selected level of shoulder elevation.
[33] 2009	Determination of the difference in fatigue between athletes and non-athletes	VL, VM, RF	11 males	Maximum versus forced repetition knee extension	Blood lactate, load in forced repetition, integrated EMG	Strength athletes produced neural fatigue in high-intensity resistance exercise.
[34] 1998	EMG assessment of back muscle function during cyclical lifting	Mind-belly of the longissimus thoracis, iliocostalis lumborum, multifidus muscles at L1, L2, L5	3 males, 1 female	Dynamic and static lifting	instantaneous median frequency (Choi–Williams)	During dynamic contractions, instantaneous median frequency behavior is nonlinear and more complex than static contraction.
[35] (2005)	Evaluation of handgrip forces using sEMG of forearm muscles	6 forearm muscles	8 males	Isometric gripping tasks	Grip force, normalized EMG	For standardized grips, valid predictions of grip based on EMG were produced.
[36] (2001)	Evaluation of the potential health effects with respect to the low back of an office chair	L3, T10	3 females, 7 males	Simulated office work on a chair	Exposure variance analysis	Trunk kinematics and erector spinae EMG were strongly affected by the task performed but not by chair type.

## References

[1] Burns A., Greene B.R., McGrath M.J., O’Shea T.J., Kuris B., Ayer S.M., Stroiescu F., Cionca V. (2010). SHIMMER™–A wireless sensor platform for noninvasive biomedical research. IEEE Sens. J..

[2] Bonato P. (2005). Advances in wearable technology and applications in physical medicine and rehabilitation. BioMed Central.

[3] Reisner A., Shaltis P., McCombie D., Asada H. A critical appraisal of opportunities for wearable medical sensors. Proceedings of the 26th Annual International Conference of the IEEE Engineering in Medicine and Biology Society.

[4] Ebashi S., Endo M., Ohtsuki I. (1969). Control of muscle contraction. Q. Rev. Biophys..

[5] Sandow A. (1952). Excitation-contraction coupling in muscular response. Yale J. Biol. Med..

[6] De Luca C.J. (1997). The use of surface electromyography in biomechanics. J. Appl. Biomech..

[7] Farina D., Merletti R., Enoka R.M. (2014). The extraction of neural strategies from the surface EMG: An update. J. Appl. Physiol..

[8] Merletti R., Parker P.J. (2004). Electromyography: Physiology, Engineering, and Non-Invasive Applications.

[9] Travell J.G. (1976). Myofascial trigger points: Clinical view. Adv. Pain Res. Ther..

[10] Chen J.-T., Chen S.-M., Kuan T.-S., Chung K.-C., Hong C.-Z. (1998). Phentolamine effect on the spontaneous electrical activity of active loci in a myofascial trigger spot of rabbit skeletal muscle. Arch. Phys. Med. Rehabil..

[11] Simons D.G., Dexter J.R. (1995). Comparison of local twitch responses elicited by palpitation and needling of myofascial trigger points. J. Musculoskelet. Pain.

[12] Campbell W.W. (2013). Essentials of Electrodiagnostic Medicine.

[13] Bunzynski T.H., Stoyva J.M., Adler C.S., Mullaney D.J. (1973). EMG biofeedback and tension headache: A controlled outcome study. Psychosom. Med..

[14] Glazer H.I., Rodke G., Swencionis C., Hertz R., Young A.W. (1995). Treatment of vulvar vestibulitis syndrome with electromyographic biofeedback of pelvic floor musculature. Obstet. Gynecol. Surv..

[15] Milner-Brown H., Stein R. (1975). The relation between the surface electromyogram and muscular force. J. Physiol..

[16] Mathiassen S., Winkel J., Hägg G. (1995). Normalization of surface EMG amplitude from the upper trapezius muscle in ergonomic studies—A review. J. Electromyogr. Kinesiol..

[17] De Luca C.J. (1984). Myoelectrical manifestations of localized muscular fatigue in humans. Crit. Rev. Biomed. Eng..

[18] Nazmi N., Abdul Rahman M.A., Yamamoto S.-I., Ahmad S.A., Zamzuri H., Mazlan S.A. (2016). A review of classification techniques of EMG signals during isotonic and isometric contractions. Sensors.

[19] Naeije M., Zorn H. (1982). Relation between EMG power spectrum shifts and muscle fibre action potential conduction velocity changes during local muscular fatigue in man. Eur. J. Appl. Physiol. Occup. Physiol..

[20] Gerdle B., Larsson B., Karlsson S. (2000). Criterion validation of surface EMG variables as fatigue indicators using peak torque: A study of repetitive maximum isokinetic knee extensions. J. Electromyogr. Kinesiol..

[21] Kollmitzer J., Ebenbichler G.R., Kopf A. (1999). Reliability of surface electromyographic measurements. Clin. Neurophysiol..

[22] Dedering Å., Németh G., Harms-Ringdahl K. (1999). Correlation between electromyographic spectral changes and subjective assessment of lumbar muscle fatigue in subjects without pain from the lower back. Clin. Biomech..

[23] Petrofsky J.S., Glaser R.M., Phillips C.A., Lind A.R., Williams C. (1982). Evaluation of the amplitude and frequency components of the surface EMG as an index of muscle fatigue. Ergonomics.

[24] Komi P.V., Tesch P. (1979). EMG frequency spectrum, muscle structure, and fatigue during dynamic contractions in man. Eur. J. Appl. Physiol. Occup. Physiol..

[25] Moritani T., Muro M., Nagata A. (1986). Intramuscular and surface electromyogram changes during muscle fatigue. J. Appl. Physiol..

[26] Mannion A.F., Dolan P. (1994). Electromyographic median frequency changes during isometric contraction of the back extensors to fatigue. Spine.

[27] Roy S.H., De Luca C.J., Schneider J. (1986). Effects of electrode location on myoelectric conduction velocity and median frequency estimates. J. Appl. Physiol..

[28] Rainoldi A., Melchiorri G., Caruso I. (2004). A method for positioning electrodes during surface EMG recordings in lower limb muscles. J. Neurosci. Methods.

[29] Ng J., Kippers V., Richardson C. (1998). Muscle fibre orientation of abdominal muscles and suggested surface EMG electrode positions. Electromyogr. Clin. Neurophysiol..

[30] Hermens H.J., Freriks B., Merletti R., Stegeman D., Blok J., Rau G., Disselhorst-Klug C., Hägg G. (1999). European recommendations for surface electromyography. Roessingh Res. Dev..

[31] Ament W., Bonga G.J., Hof A.L., Verkerke G.J. (1993). EMG median power frequency in an exhausting exercise. J. Electromyogr. Kinesiol..

[32] Minning S., Eliot C.A., Uhl T.L., Malone T.R. (2007). EMG analysis of shoulder muscle fatigue during resisted isometric shoulder elevation. J. Electromyogr. Kinesiol..

[33] Dimitrov G.V., Arabadzhiev T.I., Mileva K.N., Bowtell J.L., Crichton N., Dimitrova N.A. (2006). Muscle fatigue during dynamic contractions assessed by new spectral indices. Med. Sci. Sports Exerc..

[34] Roy S.H., Bonato P., Knaflitz M. (1998). EMG assessment of back muscle function during cyclical lifting. J. Electromyogr. Kinesiol..

[35] Hoozemans M.J., Van Dieen J.H. (2005). Prediction of handgrip forces using surface EMG of forearm muscles. J. Electromyogr. Kinesiol..

[36] Van Dieën J., De Looze M., Hermans V. (2001). Effects of dynamic office chairs on trunk kinematics, trunk extensor EMG and spinal shrinkage. Ergonomics.

[37] Akin M., Kurt M.B., Sezgin N., Bayram M. (2008). Estimating vigilance level by using EEG and EMG signals. Neural Comput. Appl..

[38] Lal S.K., Craig A. (2002). Driver fatigue: Electroencephalography and psychological assessment. Psychophysiology.

[39] Fu R., Wang H. (2014). Detection of driving fatigue by using noncontact EMG and ECG signals measurement system. Int. J. Neural Syst..

[40] Rubin D.I. (2012). Needle electromyography: Basic concepts and patterns of abnormalities. Neurol. Clin..

[41] Strommen J.A., Daube J.R. (2001). Determinants of pain in needle electromyography. Clin. Neurophysiol..

[42] Boe S.G., Stashuk D.W., Doherty T.J. (2004). Motor unit number estimation by decomposition-enhanced spike-triggered averaging: Control data, test-retest reliability, and contractile level effects. Muscle Nerve Off. J. Am. Assoc. Electrodiagn. Med..

[43] Nandedkar S.D., Barkhaus P.E., Sanders D.B., Stålberg E.V. (1988). Analysis of amplitude and area of concentric needle EMG motor unit action potentials. Electroencephalogr. Clin. Neurophysiol..

[44] Stålberg E., Falck B., Sonoo M., Stålberg S., Åström M. (1995). Multi-MUP EMG analysis—A two year experience in daily clinical work. Electroencephalogr. Clin. Neurophysiol. Electromyogr. Mot. Control.

[45] Watanabe K., Akima H. (2011). Validity of surface electromyography for vastus intermedius muscle assessed by needle electromyography. J. Neurosci. Methods.

[46] Rafiee J., Rafiee M., Yavari F., Schoen M. (2011). Feature extraction of forearm EMG signals for prosthetics. Expert Syst. Appl..

[47] Stålberg E. (2012). Jitter analysis with concentric needle electrodes. Ann. New York Acad. Sci..

[48] Ertaş M., Baslo M.B., Yildiz N., Yazici J., Öge A.E. (2000). Concentric needle electrode for neuromuscular jitter analysis. Muscle Nerve Off. J. Am. Assoc. Electrodiagn. Med..

[49] Sarrigiannis P.G., Kennett R.P., Read S., Farrugia M.E. (2006). Single-fiber EMG with a concentric needle electrode: Validation in myasthenia gravis. Muscle Nerve Off. J. Am. Assoc. Electrodiagn. Med..

[50] Finsterer J., Fuglsang-Frederiksen A., Mamoli B. (1997). Needle EMG of the tongue: Motor unit action potential versus peak ratio analysis in limb and bulbar onset amyotrophic lateral sclerosis. J. Neurol. Neurosurg. Psychiatry.

[51] Fye W.B. (1994). A history of the origin, evolution, and impact of electrocardiography. Am. J. Cardiol..

[52] Einthoven W., Fahr G., De Waart A. (1913). Über die Richtung und die manifeste Grösse der Potentialschwankungen im menschlichen Herzen und über den Einfluss der Herzlage auf die Form des Elektrokardiogramms. Pflüger’s Arch. Für Die Gesamte Physiol. Des Menschen Und Der Tiere.

[53] Kligfield P., Gettes L.S., Bailey J.J., Childers R., Deal B.J., Hancock E.W., Van Herpen G., Kors J.A., Macfarlane P., Mirvis D.M. (2007). Recommendations for the standardization and interpretation of the electrocardiogram: Part I: The electrocardiogram and its technology a scientific statement from the American Heart Association Electrocardiography and Arrhythmias Committee, Council on Clinical Cardiology; the American College of Cardiology Foundation; and the Heart Rhythm Society endorsed by the International Society for Computerized Electrocardiology. J. Am. Coll. Cardiol..

[54] Holmes J., Kubo S.H., Cody R.J., Kligfield P. (1985). Arrhythmias in ischemic and nonischemic dilated cardiomyopathy: Prediction of mortality by ambulatory electrocardiography. Am. J. Cardiol..

[55] Algra A., Tijssen J., Roelandt J., Pool J., Lubsen J. (1991). QTc prolongation measured by standard 12-lead electrocardiography is an independent risk factor for sudden death due to cardiac arrest. Circulation.

[56] Fleg J.L., Kennedy H.L. (1982). Cardiac arrhythmias in a healthy elderly population: Detection by 24-hour ambulatory electrocardiography. Chest.

[57] Wang Y., Agrafioti F., Hatzinakos D., Plataniotis K.N. (2007). Analysis of human electrocardiogram for biometric recognition. Eurasip J. Adv. Signal Process..

[58] Biel L., Pettersson O., Philipson L., Wide P. (2001). ECG analysis: A new approach in human identification. IEEE Trans. Instrum. Meas..

[59] Challoner A., Rolfe P. (1979). Non-Invasive Physiological Measurements.

[60] Kamal A., Harness J., Irving G., Mearns A. (1989). Skin photoplethysmography—A review. Comput. Methods Programs Biomed..

[61] Allen J. (2007). Photoplethysmography and its application in clinical physiological measurement. Physiol. Meas..

[62] Shelley K.H. (2007). Photoplethysmography: Beyond the calculation of arterial oxygen saturation and heart rate. Anesth. Analg..

[63] Phan D., Siong L.Y., Pathirana P.N., Seneviratne A. Smartwatch: Performance evaluation for long-term heart rate monitoring. Proceedings of the 2015 International Symposium on Bioelectronics and Bioinformatics (ISBB).

[64] Sun Y., Thakor N. (2015). Photoplethysmography revisited: From contact to noncontact, from point to imaging. Ieee Trans. Biomed. Eng..

[65] Selvaraj N., Jaryal A., Santhosh J., Deepak K.K., Anand S. (2008). Assessment of heart rate variability derived from finger-tip photoplethysmography as compared to electrocardiography. J. Med Eng. Technol..

[66] Yan Y., Poon C.C.Y., Zhang Y. (2005). Reduction of motion artifact in pulse oximetry by smoothed pseudo Wigner-Ville distribution. J. Neuroeng. Rehabil..

[67] Chan K., Zhang Y. Adaptive reduction of motion artifact from photoplethysmographic recordings using a variable step-size LMS filter. Proceedings of the SENSORS, 2002 IEEE.

[68] Seyedtabaii S., Seyedtabaii L. (2008). Kalman filter based adaptive reduction of motion artifact from photoplethysmographic signal. Int. J. Electr. Comput. Eng..

[69] Haas L.F. (2003). Hans berger (1873–1941), richard caton (1842–1926), and electroencephalography. J. Neurol. Neurosurg. Psychiatry.

[70] Teplan M. (2002). Fundamentals of EEG measurement. Meas. Sci. Rev..

[71] Al Naqeeb N., Edwards A.D., Cowan F.M., Azzopardi D. (1999). Assessment of neonatal encephalopathy by amplitude-integrated electroencephalography. Pediatrics.

[72] Anderson W.W. (1963). The hyperkinetic child: A neurological appraisal. Neurology.

[73] Casson A.J., Yates D.C., Smith S.J., Duncan J.S., Rodriguez-Villegas E. (2010). Wearable electroencephalography. IEEE Eng. Med. Biol. Mag..

[74] Huigen E., Peper A., Grimbergen C. (2002). Investigation into the origin of the noise of surface electrodes. Med Biol. Eng. Comput..

[75] Puurtinen M.M., Komulainen S.M., Kauppinen P.K., Malmivuo J.A., Hyttinen J.A. Measurement of noise and impedance of dry and wet textile electrodes, and textile electrodes with hydrogel. Proceedings of the 2006 International Conference of the IEEE Engineering in Medicine and Biology Society.

[76] Bokareva T., Hu W., Kanhere S., Ristic B., Gordon N., Bessell T., Rutten M., Jha S. Wireless sensor networks for battlefield surveillance. Proceedings of the Land Warfare Conference.

[77] Roy S.H., De Luca G., Cheng M.S., Johansson A., Gilmore L.D., De Luca C.J. (2007). Electro-mechanical stability of surface EMG sensors. Med Biol. Eng. Comput..

[78] Webster J.G. (1984). Reducing motion artifacts and interference in biopotential recording. IEEE Trans. Biomed. Eng..

[79] Agante P., De Sá J.M. ECG noise filtering using wavelets with soft-thresholding methods. Proceedings of the Computers in Cardiology.

[80] Phinyomark A., Limsakul C., Phukpattaranont P. (2009). A novel feature extraction for robust EMG pattern recognition. arXiv.

[81] Coosemans J., Hermans B., Puers R. (2006). Integrating wireless ECG monitoring in textiles. Sens. Actuators A Phys..

[82] Catrysse M., Puers R., Hertleer C., Van Langenhove L., Van Egmond H., Matthys D. (2004). Towards the integration of textile sensors in a wireless monitoring suit. Sens. Actuators A Phys..

[83] Henry S., McAllister D.V., Allen M.G., Prausnitz M.R. (1998). Microfabricated microneedles: A novel approach to transdermal drug delivery. J. Pharm. Sci..

[84] Stankovic J.A., Cao Q., Doan T., Fang L., He Z., Kiran R., Lin S., Son S., Stoleru R., Wood A. Wireless sensor networks for in-home healthcare: Potential and challenges. Proceedings of the High Confidence Medical Device Software and Systems (HCMDSS) Workshop.

[85] Ren L., Xu S., Gao J., Lin Z., Chen Z., Liu B., Liang L., Jiang L. (2018). Fabrication of flexible microneedle array electrodes for wearable bio-signal recording. Sensors.

[86] Li C.G., Lee C.Y., Lee K., Jung H. (2013). An optimized hollow microneedle for minimally invasive blood extraction. Biomed. Microdevices.

[87] Rogers J.A., Someya T., Huang Y. (2010). Materials and mechanics for stretchable electronics. Science.

[88] Song S.M., Park J.K., Sul O.J., Cho B.J. (2012). Determination of work function of graphene under a metal electrode and its role in contact resistance. Nano Lett..

[89] Kim J., Lee M.S., Jeon S., Kim M., Kim S., Kim K., Bien F., Hong S.Y., Park J.U. (2015). Highly Transparent and Stretchable Field-Effect Transistor Sensors Using Graphene–Nanowire Hybrid Nanostructures. Adv. Mater..

[90] Steinhoff G., Baur B., Wrobel G., Ingebrandt S., Offenhäusser A., Dadgar A., Krost A., Stutzmann M., Eickhoff M. (2005). Recording of cell action potentials with Al Ga N/Ga N field-effect transistors. Appl. Phys. Lett..

[91] Shao Y., Wang J., Wu H., Liu J., Aksay I.A., Lin Y. (2010). Graphene based electrochemical sensors and biosensors: A review. Electroanal. Int. J. Devoted Fundam. Pract. Asp. Electroanal..

[92] Kuzum D., Takano H., Shim E., Reed J.C., Juul H., Richardson A.G., De Vries J., Bink H., Dichter M.A., Lucas T.H. (2014). Transparent and flexible low noise graphene electrodes for simultaneous electrophysiology and neuroimaging. Nat. Commun..

[93] Park D.-W., Brodnick S.K., Ness J.P., Atry F., Krugner-Higby L., Sandberg A., Mikael S., Richner T.J., Novello J., Kim H. (2016). Fabrication and utility of a transparent graphene neural electrode array for electrophysiology, in vivo imaging, and optogenetics. Nat. Protoc..

[94] Araki T., Uemura T., Yoshimoto S., Takemoto A., Noda Y., Izumi S., Sekitani T. (2020). Wireless monitoring using a stretchable and transparent sensor sheet containing metal nanowires. Adv. Mater..

[95] Choi S., Han S.I., Jung D., Hwang H.J., Lim C., Bae S., Park O.K., Tschabrunn C.M., Lee M., Bae S.Y. (2018). Highly conductive, stretchable and biocompatible Ag–Au core–sheath nanowire composite for wearable and implantable bioelectronics. Nat. Nanotechnol..

[96] Lee S., Kim S.-W., Ghidelli M., An H.S., Jang J., Li Bassi A., Lee S.-Y., Park J.-U. (2020). Integration of Transparent Supercapacitors and Electrodes Using Nanostructured Metallic Glass Films for Wirelessly Rechargeable, Skin Heat Patches. Nano Lett..

[97] Kim J., Kim M., Lee M.-S., Kim K., Ji S., Kim Y.-T., Park J., Na K., Bae K.-H., Kim H.K. (2017). Wearable smart sensor systems integrated on soft contact lenses for wireless ocular diagnostics. Nat. Commun..

[98] Shi J., Li X., Cheng H., Liu Z., Zhao L., Yang T., Dai Z., Cheng Z., Shi E., Yang L. (2016). Graphene reinforced carbon nanotube networks for wearable strain sensors. Adv. Funct. Mater..

[99] Zhang J., Cao Y., Qiao M., Ai L., Sun K., Mi Q., Zang S., Zuo Y., Yuan X., Wang Q. (2018). Human motion monitoring in sports using wearable graphene-coated fiber sensors. Sens. Actuators A Phys..

[100] Jang J., Hyun B.G., Ji S., Cho E., An B.W., Cheong W.H., Park J.-U. (2017). Rapid production of large-area, transparent and stretchable electrodes using metal nanofibers as wirelessly operated wearable heaters. Npg Asia Mater..

[101] Määttänen A., Vanamo U., Ihalainen P., Pulkkinen P., Tenhu H., Bobacka J., Peltonen J. (2013). A low-cost paper-based inkjet-printed platform for electrochemical analyses. Sens. Actuators B Chem..

[102] Bihar E., Roberts T., Ismailova E., Saadaoui M., Isik M., Sanchez-Sanchez A., Mecerreyes D., Hervé T., De Graaf J.B., Malliaras G.G. (2017). Fully printed electrodes on stretchable textiles for long-term electrophysiology. Adv. Mater. Technol..

[103] Yeo W.H., Kim Y.S., Lee J., Ameen A., Shi L., Li M., Wang S., Ma R., Jin S.H., Kang Z. (2013). Multifunctional epidermal electronics printed directly onto the skin. Adv. Mater..

[104] Kim D.-H., Lu N., Ma R., Kim Y.-S., Kim R.-H., Wang S., Wu J., Won S.M., Tao H., Islam A. (2011). Epidermal electronics. Science.

[105] Lipomi D.J., Vosgueritchian M., Tee B.C., Hellstrom S.L., Lee J.A., Fox C.H., Bao Z. (2011). Skin-like pressure and strain sensors based on transparent elastic films of carbon nanotubes. Nat. Nanotechnol..

[106] Segev-Bar M., Haick H. (2013). Flexible sensors based on nanoparticles. ACS Nano.

[107] Unno Y., Affolder A., Allport P., Bates R., Betancourt C., Bohm J., Brown H., Buttar C., Carter J., Casse G. (2011). Development of n-on-p silicon sensors for very high radiation environments. Nucl. Instrum. Methods Phys. Res. Sect. A Accel. Spectrometersdetectors Assoc. Equip..

